# Supplementary material for: Trapping a somatic endogenous retrovirus into a germline piRNA cluster immunizes the germline against further invasion
Source: Genome Biol. 2019 Jun 21;20:127. doi: 10.1186/s13059-019-1736-x (PMC6587276; doi:10.1186/s13059-019-1736-x)
Supplement: Supplementary file 1 — Figure S1. The RevI-H2 line carries a deletion that removes ZAM from the flamenco piRNA cluster. Figure S2. In RevI-H2 ovaries, piRNAs derived from Burdock, the prototypic germinal TE, present similar features as those derived from ZAM. Figure S3. ZAM-derived piRNAs are de novo produced by the germline of RevI-H2 ovaries. Figure S4. ZAM-derived piRNAs originate from a germline piRNA cluster localized on the X chromosome. Figure S5. Phidippo- and Pifo-derived piRNAs are mainly produced by the flamenco cluster. Figure S6. ZAM-derived piRNAs produced in the different Rev lines display similar features. (PDF 3179 kb) [file 13059_2019_1736_MOESM1_ESM.pdf]

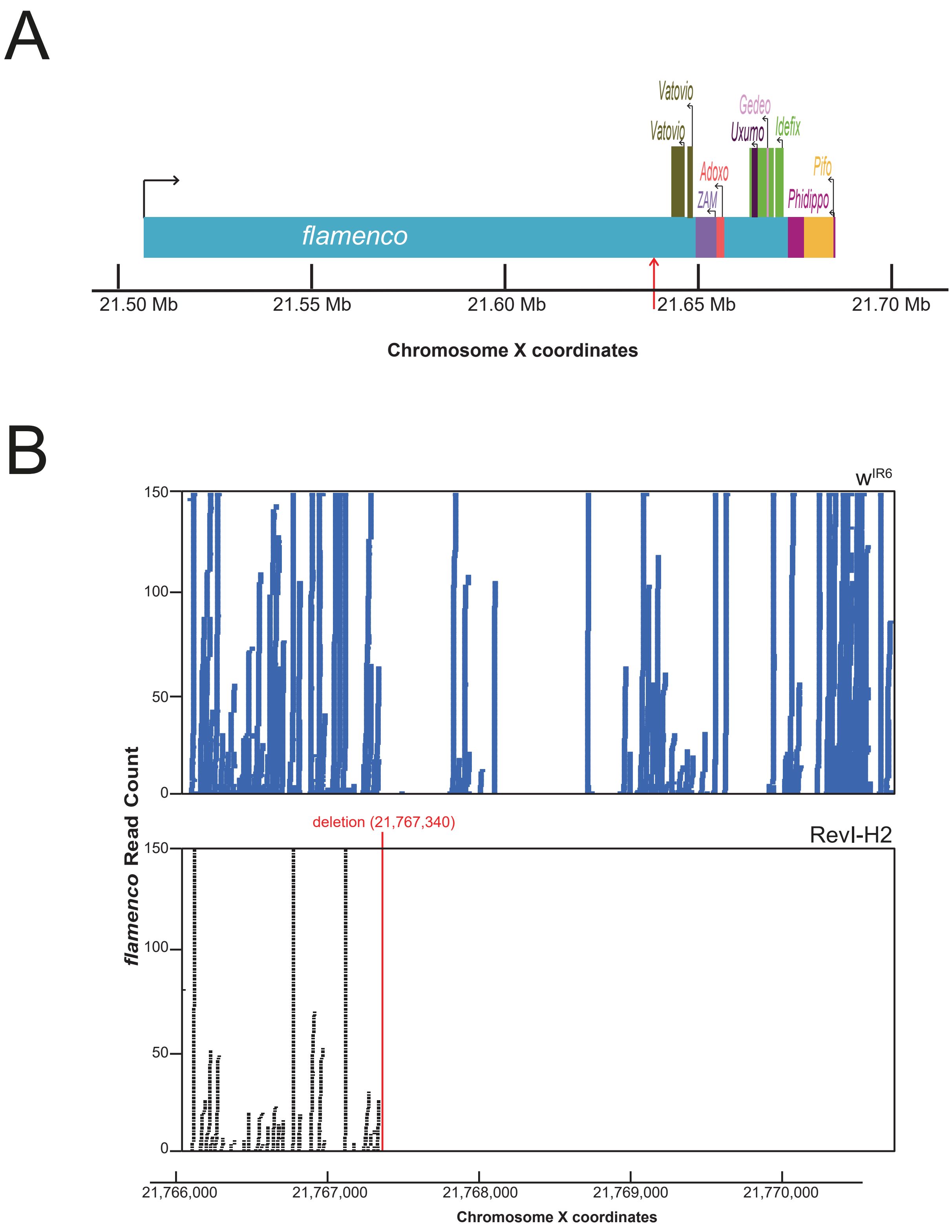

Figure S1. The RevI-H2 line carries a deletion that removes *ZAM* from the *flamenco* piRNA cluster.
a Structure of the *flamenco* piRNA cluster in *D. melanogaster*. TEs located in the region of *flamenco* deleted in RevI-H2 are presented individually above *flamenco* according to [26]. The centromere of the X chromosome is on the right-hand (proximal) side. Sense-strand transcription for *flamenco* and TE orientation are indicated by black arrows. The *flamenco* deletion distal break-point in RevI-H2 [26] is indicated by a red arrow. The chromosome coordinates are according to release 5 of the *D. melanogaster* genome. **b** Genome browser panel showing *flamenco* piRNA levels in w^{IR6} and RevI-H2 line. The refined Release 6.03 coordinate for the break-point of the RevI-H2 deletion in *flamenco* reported in [26] is displayed by a red line.

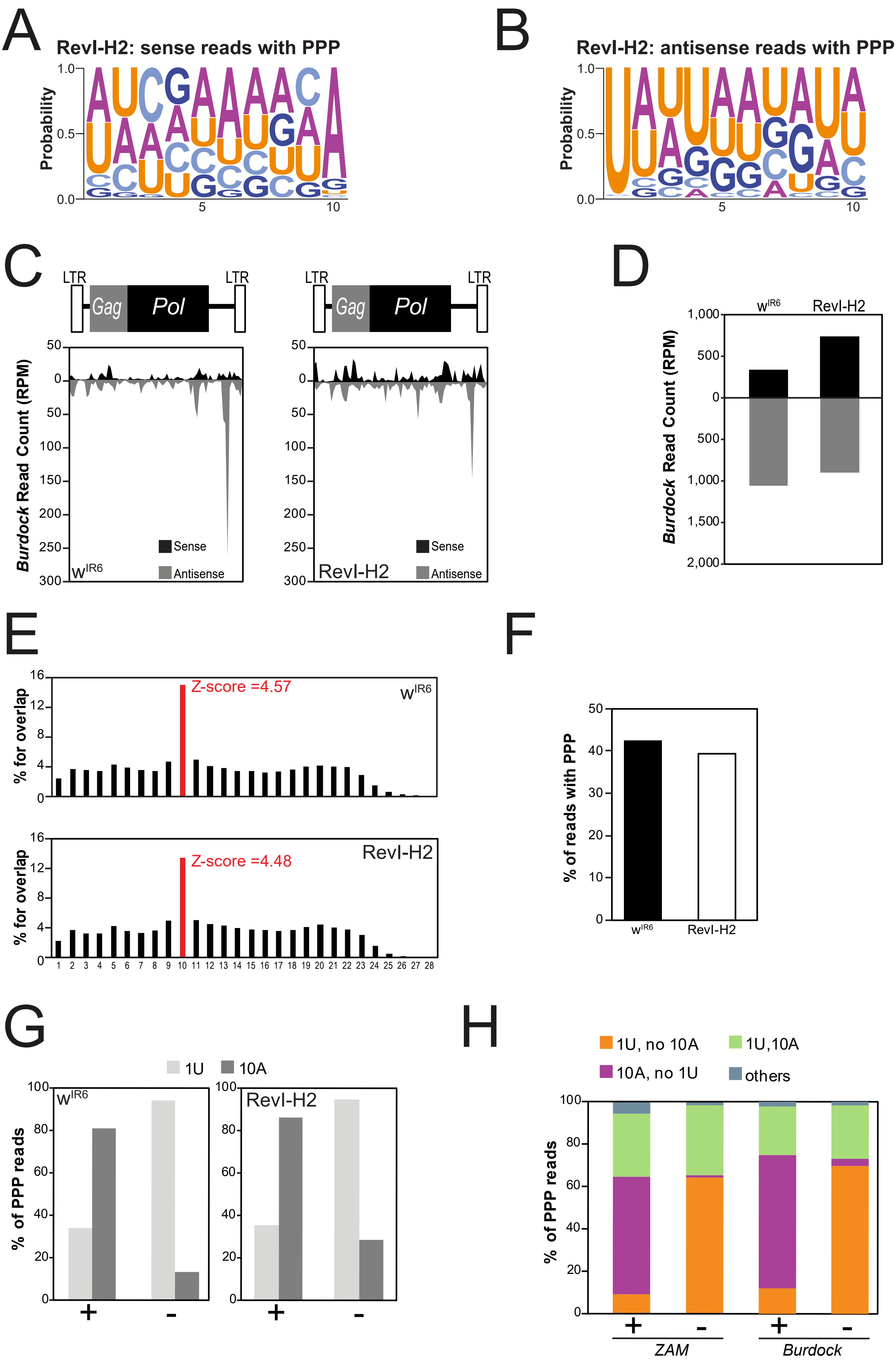
A
RevI-H2: sense reads with PPP
Probability
1.0
0.5
0.0
5
10
B
RevI-H2: antisense reads with PPP
Probability
C
LTR
Gag
Pol
LTR
Burdock Read Count (RPM)
50
0
50
100
150
200
250
300
Sense
Antisense
wIR6
RevI-H2
D
wIR6
RevI-H2
Burdock Read Count (RPM)
1,000
500
0
500
1,000
1,500
2,000
E
% for overlap
16
12
8
4
0
Z-score =4.57
wIR6
Z-score =4.48
RevI-H2
1 2 3 4 5 6 7 8 9 10 11 12 13 14 15 16 17 18 19 20 21 22 23 24 25 26 27 28
F
% of reads with PPP
50
40
30
20
10
0
wIR6
RevI-H2
G
1U
10A
% of PPP reads
100
80
60
40
20
0
wIR6
RevI-H2
+
-
H
1U, no 10A
1U,10A
10A, no 1U
others
% of PPP reads
100
80
60
40
20
0
+
-
+
-
ZAM
Burdock

Figure S2. In RevI-H2 ovaries, piRNAs derived from *Burdock*, the prototypic germinal TE, present similar features as those derived from *ZAM*.

a-b Logo of nucleotide bias for the first ten positions of sense (*a*) and antisense (*b*) *ZAM*-derived piRNAs with ping-pong partner (PPP) produced in RevI-H2 ovaries. The nucleotide height represents its relative frequency at that position. **c** Density profile of *Burdock*-derived piRNAs along the 6.4kb *Burdock* sequence in w^{IR6} (left) and RevI-H2 (right) ovaries (all mapping piRNAs allowing up to 3 mismatches). Sense and antisense reads are in black and grey, respectively. The organization of *Burdock* is displayed above the profiles. **d** The total amount of *Burdock*-derived piRNAs produced in w^{IR6} and RevI-H2 ovaries was quantified from the profiles in *c*. **e** Histogram showing the percentage of 5'-overlaps between sense and antisense *Burdock*-derived piRNAs (23-29nt) in w^{IR6} (top) and RevI-H2 (bottom) ovaries. The peak in red defines the proportion of 10nt-overlapping pairs and the Z-score is indicated. **f** Bar diagram indicating the percentage of *Burdock*-derived piRNAs with ping-pong partner (PPP) in the w^{IR6} and RevI-H2 lines. **g** Analysis of the nucleotide bias for sense (+) and antisense (-) *Burdock*-derived piRNAs with PPP in w^{IR6} (left) and RevI-H2 (right). The percentages of PPPs with a 1U and 10A are displayed. **h** Analysis of nucleotide bias for sense (+) and antisense (-) *ZAM*- and *Burdock*-derived piRNAs with PPPs in RevI-H2 ovaries.

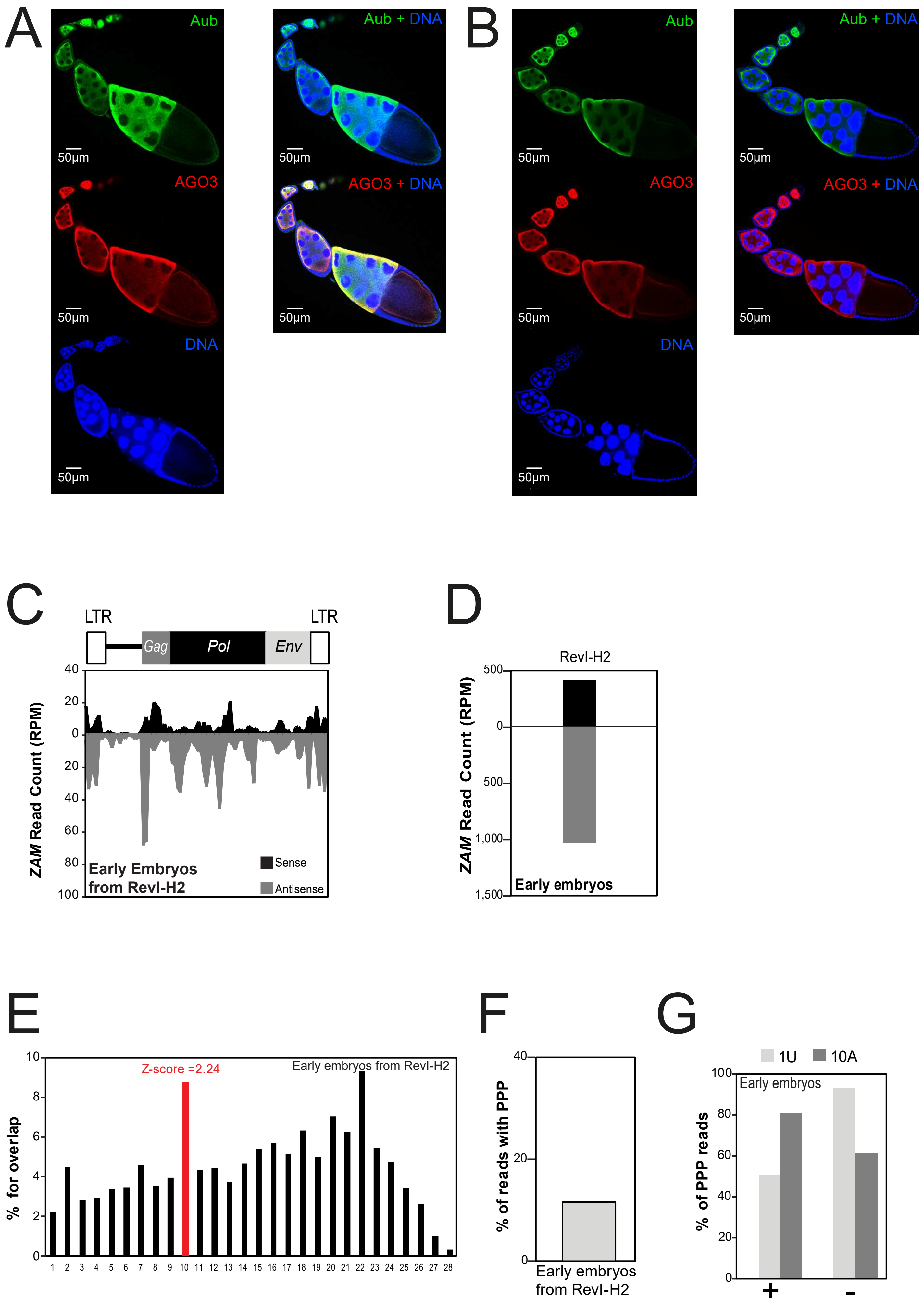
A
Aub
Aub + DNA
AGO3
AGO3 + DNA
DNA
50µm
B
Aub
Aub + DNA
AGO3
AGO3 + DNA
DNA
50µm
C
LTR
LTR
Gag
Pol
Env
ZAM Read Count (RPM)
Early Embryos from RevI-H2
Sense
Antisense
D
RevI-H2
ZAM Read Count (RPM)
Early embryos
E
Z-score =2.24
Early embryos from RevI-H2
% for overlap
F
% of reads with PPP
Early embryos from RevI-H2
G
1U
10A
Early embryos
% of PPP reads
+
-

H

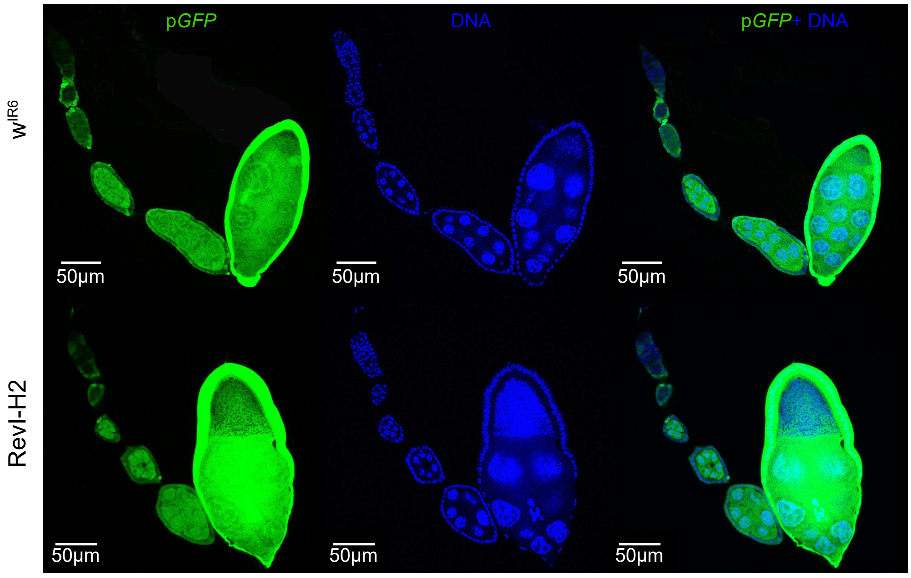
pGFP
DNA
pGFP+ DNA
wIR6
RevI-H2
50µm

I

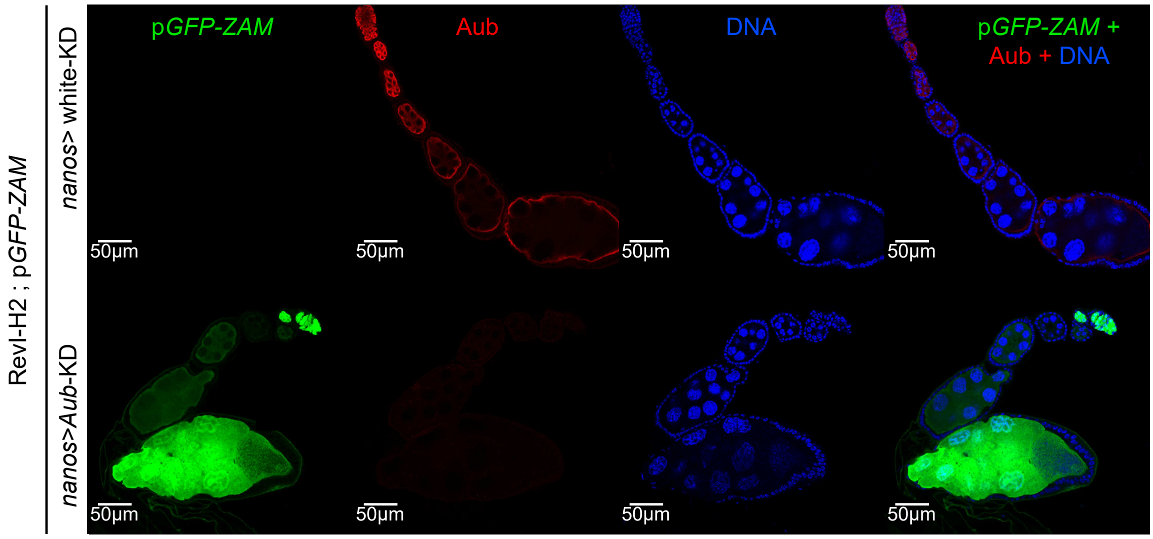
pGFP-ZAM
Aub
DNA
pGFP-ZAM + Aub + DNA
RevI-H2 ; pGFP-ZAM
nanos> white-KD
nanos>Aub-KD
50µm

J

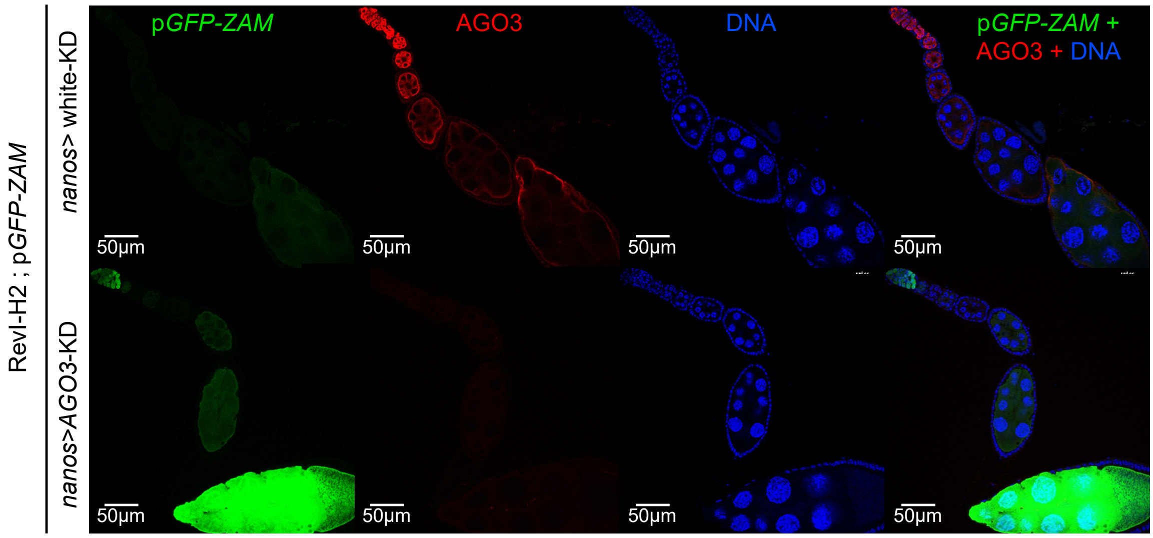
pGFP-ZAM
AGO3
DNA
pGFP-ZAM + AGO3 + DNA
RevI-H2 ; pGFP-ZAM
nanos> white-KD
nanos>AGO3-KD
50µm

Figure S3. *ZAM*-derived piRNAs are *de novo* produced by the germline of RevI-H2 ovaries.
a-b Confocal images of ovarioles from w^{IR6} (*a*) and RevI-H2 (*b*) ovaries after labeling with anti-Aub (green, top) and -Ago3 (red, middle) antibodies and DNA (blue, bottom) staining. Merged images of the Aub or Ago3 signal and DNA staining are displayed on the right panels. **c** Density profile of *ZAM*-derived piRNAs along the *ZAM* sequence produced in early embryos from RevI-H2 (allowing up to 3 mismatches). Sense and antisense reads are represented in black and grey, respectively. *ZAM* organization is displayed above the profile. **d** The total amount of *ZAM*-derived piRNAs produced in early embryos from RevI-H2 was quantified from the profile in Fig. S3C. Sense and antisense reads are represented in black and grey, respectively. **e** Histogram showing the percentage of 5'-overlaps between sense and antisense *ZAM*-derived piRNAs (23-29nt) in early embryos from RevI-H2. The peak in red defines the proportion of 10nt-overlapping pairs and the Z-score is indicated. **f** Bar diagram indicating the percentage of *ZAM*-derived piRNAs with ping-pong partner (PPP) in early RevI-H2 embryos. **g** Analysis of the nucleotide bias for sense (+) and antisense (-) *ZAM*-derived piRNAs with PPPs in early RevI-H2 embryos. The percentages of PPPs with a 1U and those with a 10A are displayed. **h** Confocal images of ovarioles after GFP (green, left panels) and DNA (blue, middle panels) staining. Ovarioles were from the progeny of a cross between w^{IR6} or RevI-H2 females and males carrying the p*GFP-ZAM* sensor transgene – obtained after excision of the ZAM sequence upon recombination between the flanking FRTs – driven by *actin*-Gal4. Right panels, merged images of GFP and DNA labeling. **i-j** Confocal images of ovarioles after GFP (green), Aub (red, *i*) or AGO3 (red, *j*) and DNA (blue) staining. Ovarioles were from the progeny of a cross between RevI-H2 females carrying the *nanos*-Gal4 driver and males carrying the p*GFP* sensor transgene with either of the RNAi *Aub*- or *AGO3*-KD (Knock-Down). The white-KD was used as a control. Right panels, merged images of GFP, Aub (*i*) or AGO3 (*j*) and DNA labeling.

A

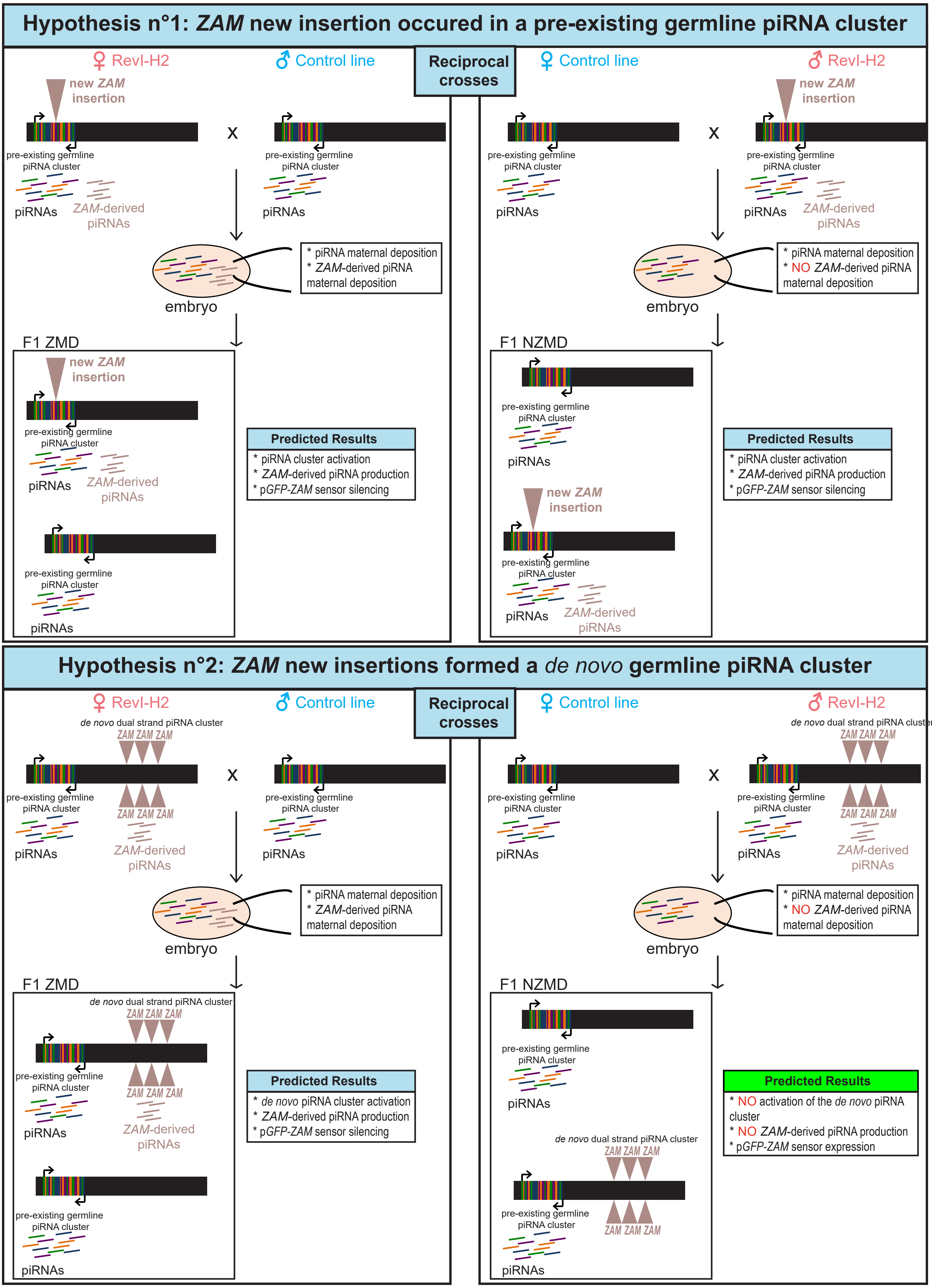
Hypothesis n°1: ZAM new insertion occured in a pre-existing germline piRNA cluster
♀ RevI-H2
♂ Control line
Reciprocal crosses
♀ Control line
♂ RevI-H2
new ZAM insertion
x
pre-existing germline piRNA cluster
piRNAs
ZAM-derived piRNAs
embryo
* piRNA maternal deposition
* ZAM-derived piRNA maternal deposition
* piRNA maternal deposition
* NO ZAM-derived piRNA maternal deposition
F1 ZMD
F1 NZMD
Predicted Results
* piRNA cluster activation
* ZAM-derived piRNA production
* pGFP-ZAM sensor silencing
Predicted Results
* piRNA cluster activation
* ZAM-derived piRNA production
* pGFP-ZAM sensor silencing
Hypothesis n°2: ZAM new insertions formed a de novo germline piRNA cluster
de novo dual strand piRNA cluster
ZAM ZAM ZAM
Predicted Results
* de novo piRNA cluster activation
* ZAM-derived piRNA production
* pGFP-ZAM sensor silencing
Predicted Results
* NO activation of the de novo piRNA cluster
* NO ZAM-derived piRNA production
* pGFP-ZAM sensor expression

B

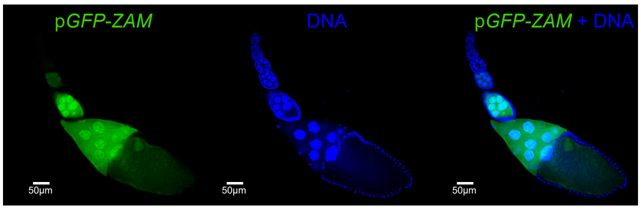

F1 progeny (cross female w^{IR6} with male p*GFP-ZAM*)

C

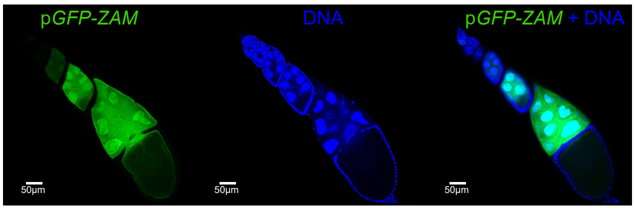

F1 progeny (cross male w^{IR6} with female p*GFP-ZAM*)

D

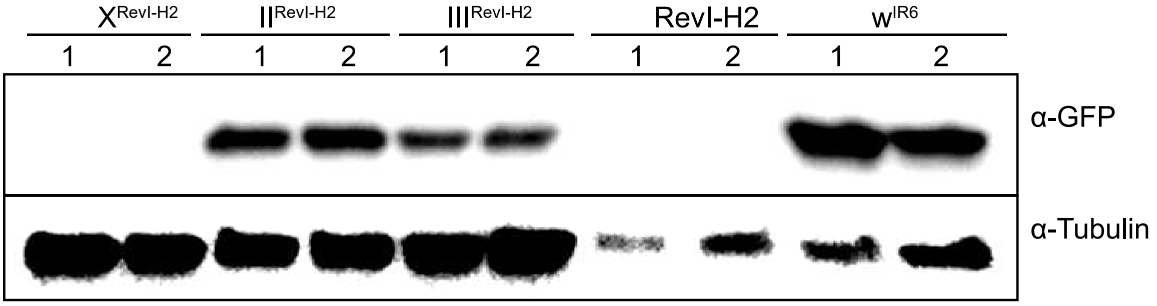

E

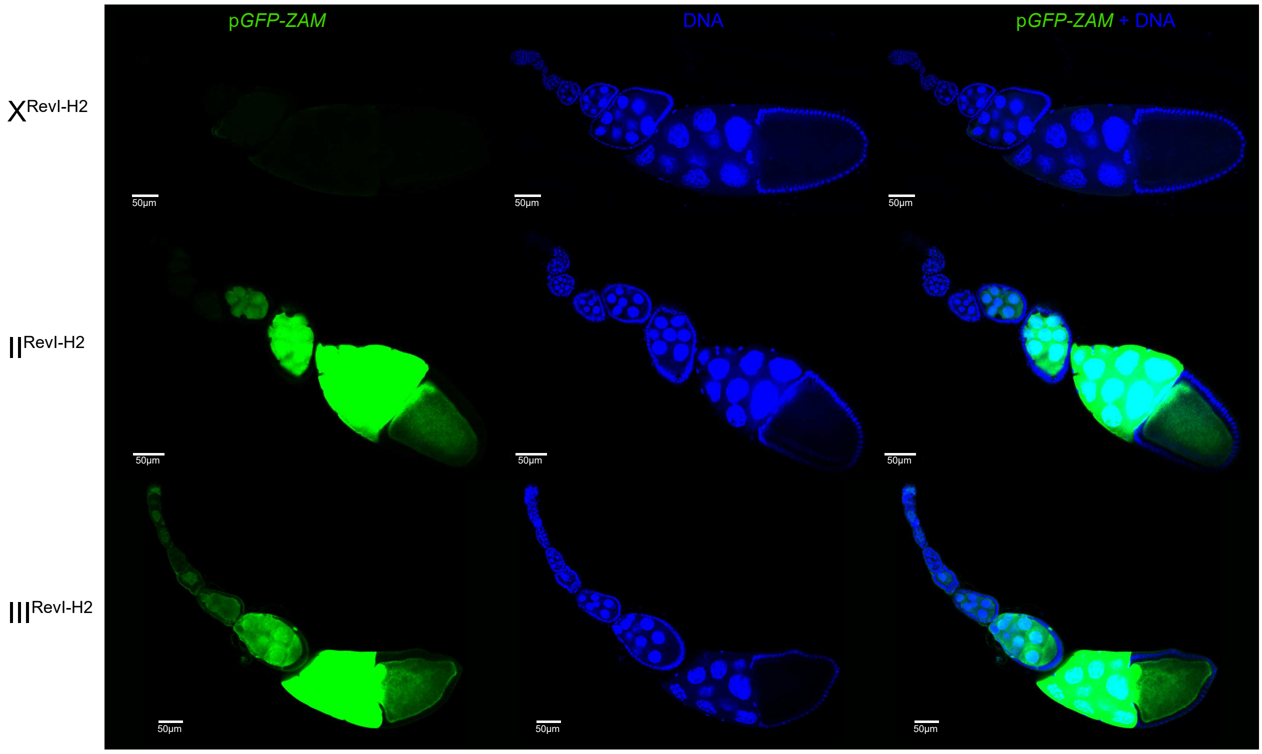

F

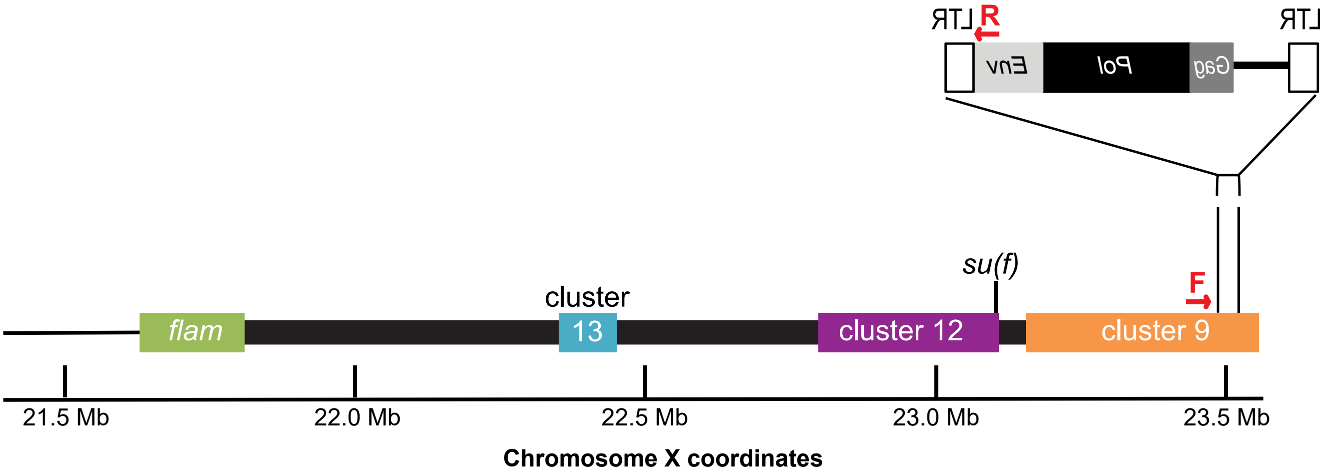

LTR
R
Env
Pol
Gag
LTR
su(f)
F
flam
cluster
13
cluster 12
cluster 9
21.5 Mb
22.0 Mb
22.5 Mb
23.0 Mb
23.5 Mb
Chromosome X coordinates

G

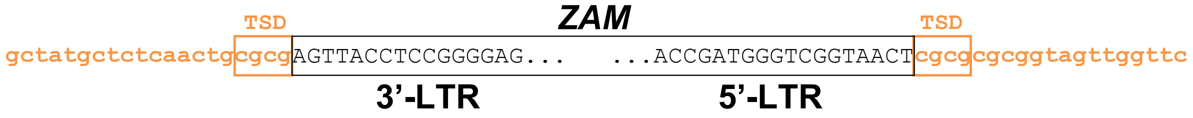

TSD
ZAM
TSD
gctatgctctcaactgcgcgAGTTACCTCCGGGGAG... ...ACCGATGGGTCGGTAACTcgcgcgcggtagttggttc
3'-LTR
5'-LTR

H

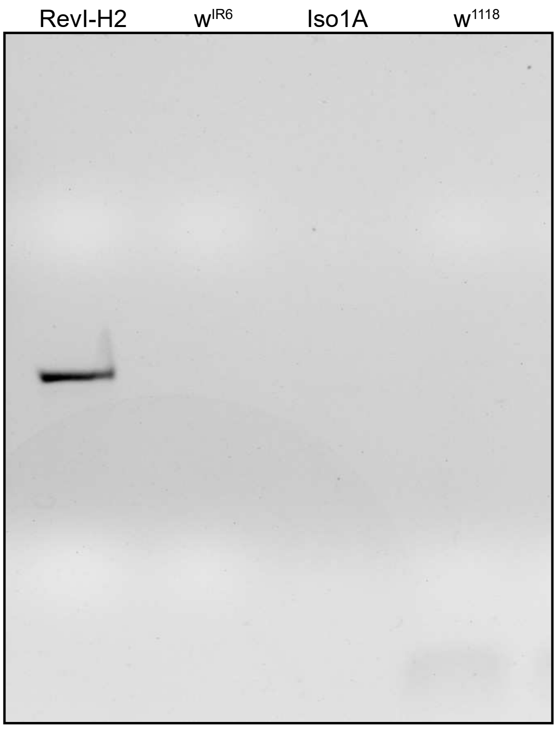

RevI-H2
wIR6
Iso1A
w1118

I

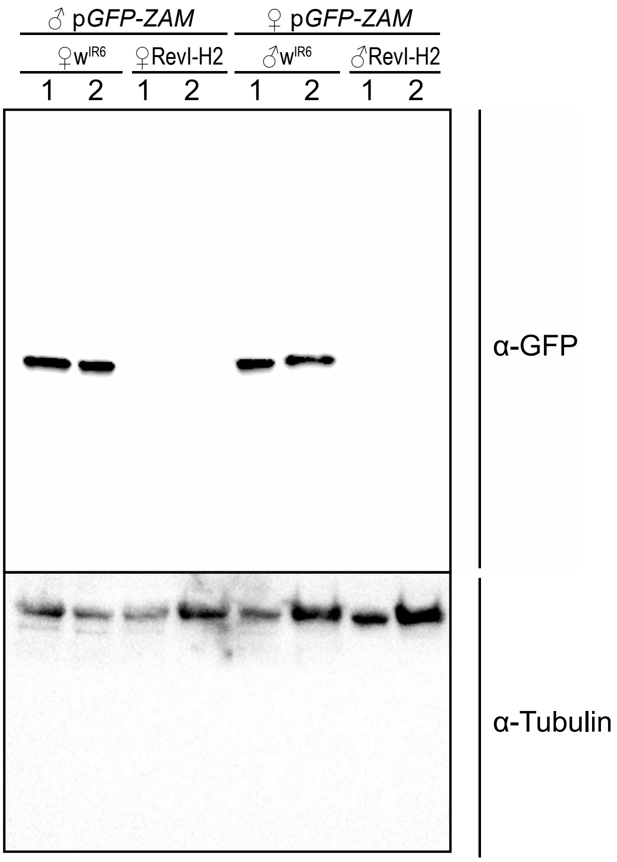

♂ pGFP-ZAM
♀ pGFP-ZAM
♀wIR6
♀RevI-H2
♂wIR6
♂RevI-H2
1 2 1 2 1 2 1 2
α-GFP
α-Tubulin

Figure S4. *ZAM*-derived piRNAs originate from a germline piRNA cluster localized on the X chromosome.

a Schema representing crosses performed to generate the ZMD and NZMD F1 progeny analyzed in Figure 4. The schema details both hypothesis for the new *ZAM* insertion in the RevI-H2 line. Only the X chromosome is displayed on the schema. The control line is the p*GFP-ZAM* line in which expression is driven in germline cells by a nanos-Gal4 driver. **b-c** Confocal images of ovarioles after staining for GFP (green, left panels) and DNA (blue, middle panels). Merged images of the GFP and DNA signals are on the right. Ovarioles were from the progeny of a cross between w^{IR6} females and control males (*b*) and from the reciprocal cross between wIR6 males and control females (*c*). In both crosses, the p*GFP-ZAM* line in which expression is driven in germline cells by a *nanos*-Gal4 driver was the control line. **d** Western blot analysis of proteins extracted from ovaries of progenies of crosses between X^{Rev}, II^{Rev} or III^{Rev} females with control males. The control line was the same as in *b* and *c*. Proteins were from two biological replicates (1&2) prepared from 5 pairs of ovaries and α-tubulin was the loading control. **e** Confocal images of ovarioles after staining for GFP (green, left panels) and DNA (blue, middle panels). Merged images of the GFP and DNA signals are on the right. Ovarioles were from the progeny of a cross between X^{Rev}, II^{Rev} or III^{Rev} females with control males. The control line was the same as in *b* and *c*. **f** Structure of the region where the new *ZAM* insertion was identified in RevI-H2. The insertion is located in the dual-strand piRNA cluster 9 (according to ranking of piRNA clusters identified in *D. melanogaster* by [30]). *ZAM* is in genomic minus strand orientation. The chromosome coordinates are according to release 6 of the *D. melanogaster* genome. The su(f) genetic marker is indicated. The pericentromeric part of the X chromosome is displayed by a black box, euchromatin as a black line, *flam* and the other piRNA clusters by colored boxes. Primers (F and R) used to confirm the presence of the *ZAM* insertion by PCR (Fig. S4H) are depicted by red arrows. The *ZAM* insertion was localized in region X: 23,474,449..23,513,109 by genome sequencing of RevI-H2 (3 identical possible insertion sites). **g** The sequenced junctions between *ZAM* and the genomic flanks. The *ZAM* sequence is displayed in black capital letter and the genomic flanking sequence matching to cluster 9 in orange lower case letters. Target site duplications (TSD) are boxed. **h** PCR analysis of the new *ZAM* insertion identified in RevI-H2. Used primers are displayed in Fig. S4F. Only RevI-H2 presents an amplicon showing that the w^{IR6}, Iso1A and w^{1118} lines are devoid of this insertion and confirming the presence of this *ZAM* insertion located in cluster 9 in RevI-H2. **i** Western blotting of proteins extracted from ovaries of progenies of crosses between w^{IR6} or RevI-H2 and the same control line as in Fig. 4*a*. The lines used for the crosses are indicated above. Proteins were from two biological replicates (1&2) prepared from 5 pairs of ovaries; α-tubulin was used as loading control. Images displayed in this panel are the unproccessed versions of those displayed in Fig. 4c.

A

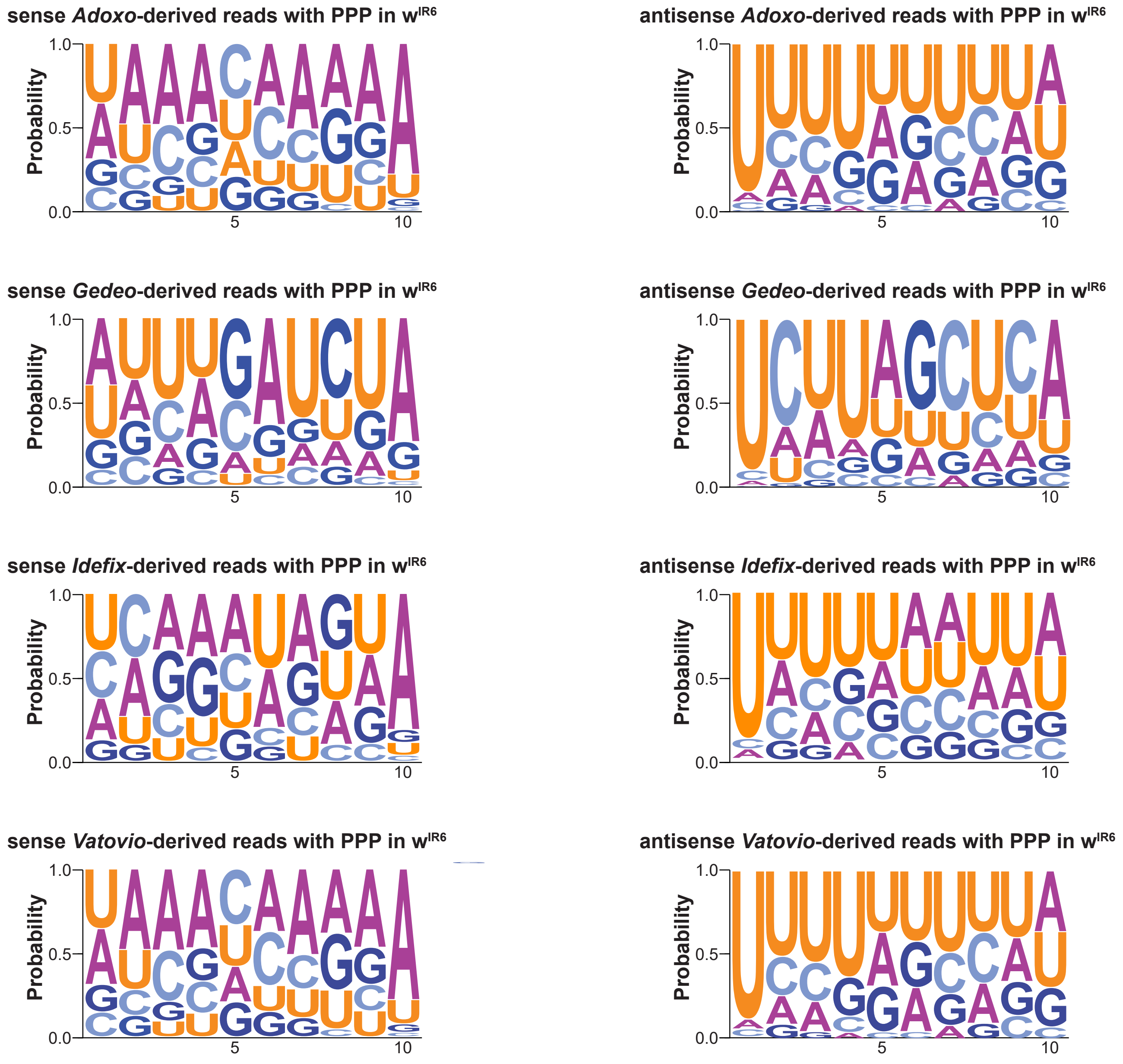
sense *Adoxo*-derived reads with PPP in w^{IR6}
antisense *Adoxo*-derived reads with PPP in w^{IR6}
sense *Gedeo*-derived reads with PPP in w^{IR6}
antisense *Gedeo*-derived reads with PPP in w^{IR6}
sense *Idefix*-derived reads with PPP in w^{IR6}
antisense *Idefix*-derived reads with PPP in w^{IR6}
sense *Vatovio*-derived reads with PPP in w^{IR6}
antisense *Vatovio*-derived reads with PPP in w^{IR6}
Probability

B

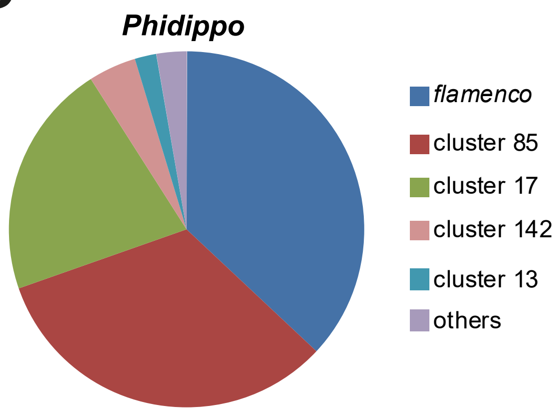
Phidippo
flamenco
cluster 85
cluster 17
cluster 142
cluster 13
others

C

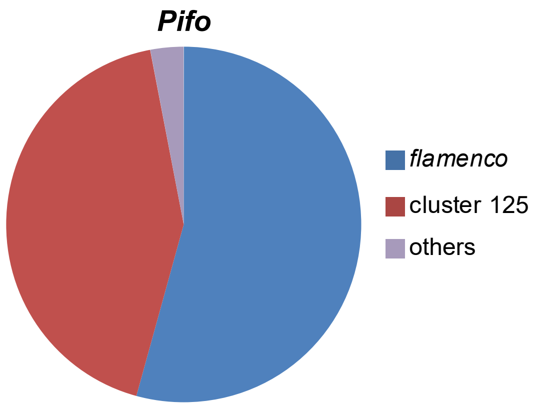
Pifo
flamenco
cluster 125
others

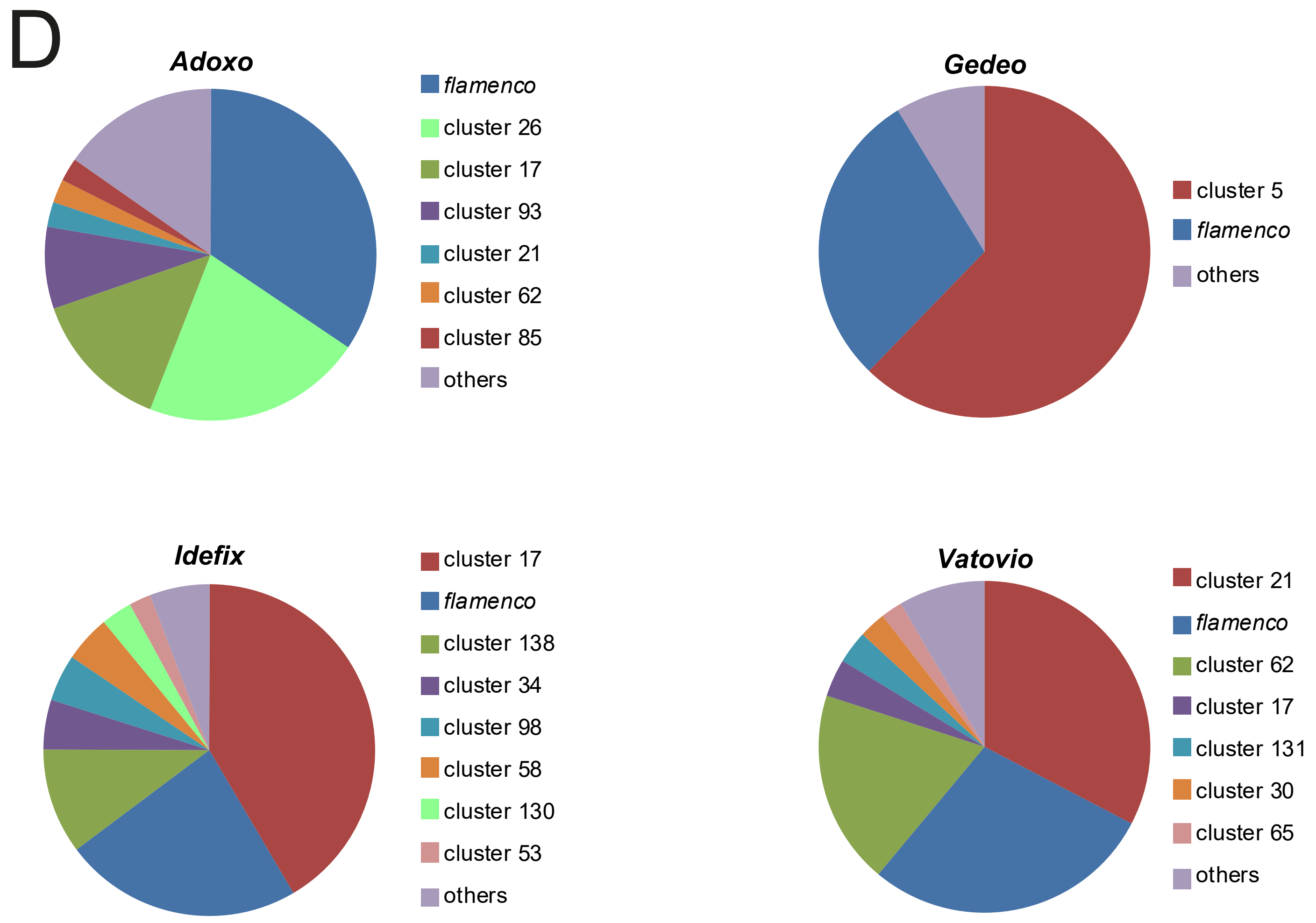

Figure S5. *Phidippo-* and *Pifo*-derived piRNAs are mainly produced by the *flamenco* cluster.
a Logo of nucleotide bias for the first ten positions of *Adoxo-, Gedeo-, Idefix-* and *Vatovio*-derived piRNAs with ping-pong partner (PPP) produced in w^{IR6} ovaries. The nucleotide height represents its relative frequency at that position. **b-c** Pie charts showing the proportion of *Phidippo-* (*a*) and *Pifo*-derived piRNAs (*b*) mapped (allowing up to 3 mismatches) to the 142 piRNA clusters (allowing no mismatch, piRNA clusters as in [9]) in the w^{IR6} line. **d** Pie charts showing the proportion of A*doxo-, Gedeo-, Idefix-* and *Vatovio*-derived piRNAs mapped (allowing up to 3 mismatches) to the 142 piRNA clusters (allowing no mismatch, piRNA clusters as in [9]) in the w^{IR6} line.

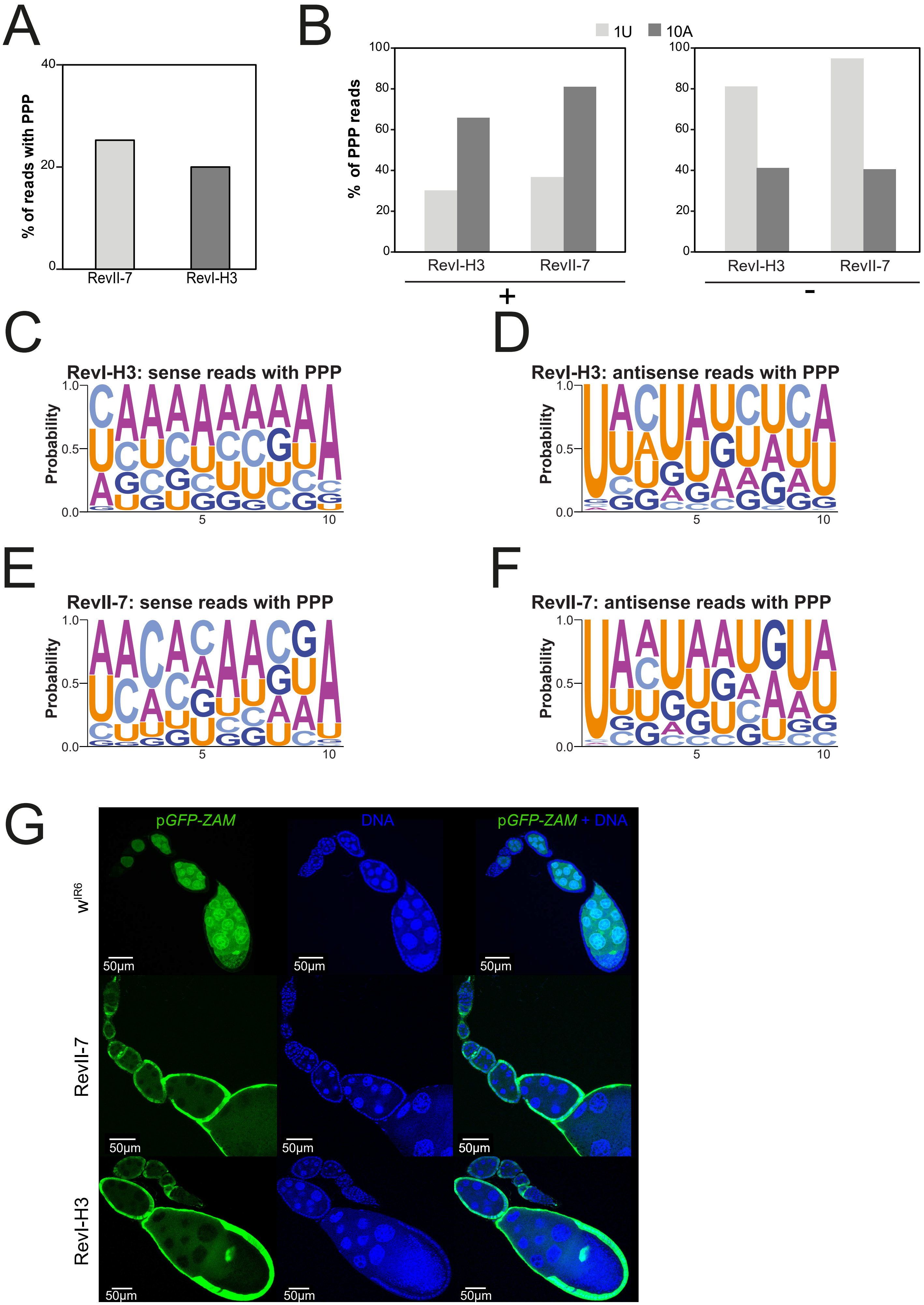
A
% of reads with PPP
40
20
0
RevII-7
RevI-H3
B
1U
10A
% of PPP reads
100
80
60
40
20
0
RevI-H3
RevII-7
+
-
C
RevI-H3: sense reads with PPP
D
RevI-H3: antisense reads with PPP
E
RevII-7: sense reads with PPP
F
RevII-7: antisense reads with PPP
Probability
1.0
0.5
0.0
5
10
G
pGFP-ZAM
DNA
pGFP-ZAM + DNA
w^IR6
RevII-7
RevI-H3
50μm

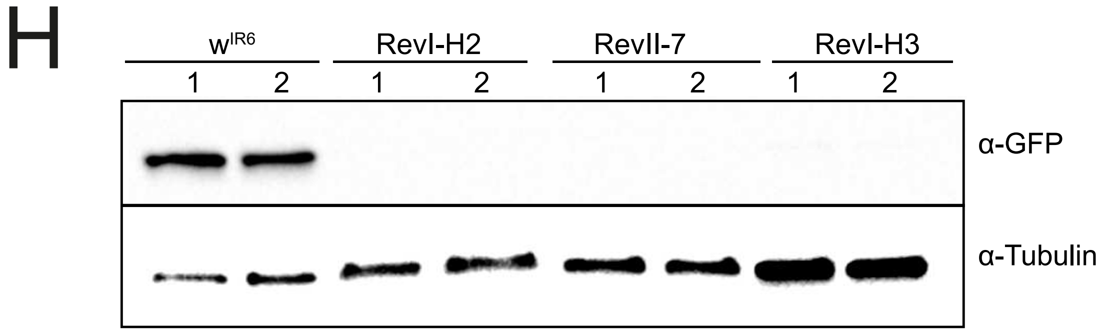

Figure S6. *ZAM*-derived piRNAs produced in the different Rev lines display similar features.
a Percentage of *ZAM*-derived piRNAs with ping-pong partner (PPP) in the RevII-7 and RevI-H3 lines. **b** Analysis of the nucleotide bias for sense (+) and antisense (-) ZAM-derived piRNAs with PPPs in the RevII-7 and RevI-H3 lines. The percentages of PPPs with a 1U and a 10A are shown. Both lines had a 10A bias for sense piRNAs and a 1U bias for antisense piRNAs. **c-f** Logo of nucleotide bias for the first ten positions of sense (*c*, *e*) and antisense (*d, f*) *ZAM*-derived piRNAs with ping-pong partner (PPP) produced in RevI-H3 (*c-d*) and RevII-7 (*e-f*) ovaries. The nucleotide height represents its relative frequency at that position. **g** Confocal images of ovarioles after staining for GFP (green, left panels) and DNA (blue, middle panels). Merged images of the GFP and DNA signals are on the right. Ovarioles were from the progeny of crosses between w^{IR6} (top), RevII-7 (middle) or RevI-H3 (bottom) females with males that harbor the p*GFP-ZAM* sensor transgene driven by the *actin*-Gal4 driver. **h** Western blot analysis of proteins extracted from ovaries of progenies of crosses between w^{IR6}, RevI-H2, RevII-7 or RevI-H3 females with a control male. The p*GFP-ZAM* line in which *ZAM* expression is driven in germline cells by a *nanos*-Gal4 driver was the control line. Proteins were from two biological replicates (1&2) prepared from 5 pairs of ovaries and α-tubulin was the loading control.
