# Supplementary material for: Trapping a somatic endogenous retrovirus into a germline piRNA cluster immunizes the germline against further invasion
Source: Genome Biol. 2019 Jun 21;20:127. doi: 10.1186/s13059-019-1736-x (PMC6587276; doi:10.1186/s13059-019-1736-x)
Supplement: Supplementary file 2 — Review history. (DOCX 515 kb) [file 13059_2019_1736_MOESM2_ESM.docx]

**First round of review**

**Reviewer 1**

In their paper, Duc et al. investigate how Drosophila germline cells respond to the introduction of somatic follicular cell-specific ZAM endogenous retrovirus. The experiments are based on the observation that in Drosophila lines harboring a deletion within flamenco, a major piRNA cluster that is specifically expressed in somatic follicular cells, ZAM is de-repressed and invades germline genome. In the mutants, ZAM elements are inserted into a germline dual-strand piRNA cluster and corresponding piRNAs with the ping-pong signature are produced to silence ZAM in germline cells. These findings suggest that the germline can protect itself by trapping invading somatic-specific transposable elements (TEs) into germline piRNA clusters.

The mechanisms of transposon silencing in germ cells and silencing of invading genetic elements are very important for fertility and evolutionary history, yet they are incompletely understood. The Duc et al. study provides insight into a long-standing question of how invading somatic TEs are acted upon in the ovaries. The manuscript is of general interest and thus well within the scope of Genome Biology.

General remarks:
1. This work lacks information as to how germ cells initially respond to ZAM invasion and what might be the consequences of ZAM invasion and then how germ cells recover from the damage caused by the invasion. The RevI-H2 line the authors use in the paper was established nearly twenty years ago. 

2. The authors conclude that ZAM-derived piRNAs are originated from a new ZAM insertion into a germline dual-strand piRNA cluster mainly because they display a ping-pong signature. However, strong evidence to support the conclusion is not present in the paper. The authors shall consider sequencing the RevI-H2 genome to identify ZAM insertions in germline piRNA clusters.

3. The novelty of the present study has been somewhat dampened by previous studies by others including Theurkauf, Zhao Zhang and their colleagues examining how the host germ cells initially respond to an invasion and subsequently tame TEs. The authors should cite their papers (Khurana et al., Cell 2011; Moon et al., Dev Cell 2018) and discuss their work. 

Some other comments:
4. Figure 3F, 6B, and 6E do not appear to show a bias for adenine at the tenth position (10A). 

5. It would be kind to the reader if Figure 2B indicated transcription direction and size scale.

6. References are rough and sloppy. For example, many of papers in References contain information on publishers but many others do not. 

7. The authors should define "PPP" in the main text but not in figure legend.

**Reviewer 2**

In this manuscript, Duc et al describe an interesting case in which the retrotransposon ZAM is trapped into a germline cluster, leading to the production of ping-pong derived piRNAs, presumably to protect te germline from further ZAM integrations. This work fits the hypothesis that piRNA clusters are a repository of past transposon invasions that provide piRNA-based protection from transposon activity/mobility. In this model, new transposon invasions would be uncontrolled until an insertion into a piRNA clusters initiates a piRNA-based responses. Although this model is widely accepted, it has been hard to capture the event of novel transposon insertions into clusters. Yet, there is some experimental evidence for this model, also in flies (e.g. Khurana et al Cell 2011, which to my surprise was not discussed in the current manuscript). 
The current manuscript differs from previous work in that ZAM is a somatic transposon that is repressed in somatic follicular cells by the flamenco locus. The authors study a fly line in which part of the flamenco locus is deleted, including the part that produces ZAM piRNAs, which lead to ZAM invasion of the germline. Hence, the transposon invasion in this manuscript is from an endogenous somatic source.
I found this an well-written and interesting manuscript. See below for my comments and suggestions. 

Major points:
1. Support for a new ZAM insertion is rather indirect and mostly based on genetics, through the detection of ZAM-derived ping-pond dependent piRNAs and repression of a GFP-ZAM transgene. The manuscript would be stronger if the authors could show direct evidence of a new ZAM insertion. As the authors propose that the insertion occurred in a pre-existing cluster (on chromosome X), a candidate PCR based approach or inverse PCR may work, or otherwise a deep-sequencing based approach could be explored. This would also help to define whether the insertions in the different Rev lines (page 12) are similar or different. 
2. Several instances quantitative claims are made based on deep sequencing data, which is not a reliable quantitative method. As far as I see, the small RNA analyses are derived from a single library and thus prone to biological noise (even if the libraries are made from material from multiple animals). Multiple independent small RNA libraries should be analysed anywhere quantative conclusions are drawn. 
3. Page 9, the authors propose that ZAM could have inserted into an pre-existing cluster or could form a novel cluster. However, the experiment performed does not directly discriminate between both possibilities, yet a strong conclusion is made that ZAM inserted into a pre-existing cluster (line 47). Directly mapping the new insertion (see point 1) would address this concern. Should that not be successful, the statement needs to be adjusted. 
4. Fig.1 . A control experiment with a GFP transgene without a ZAM sequence in the UTR would be needed as a reference. 

Minor points
Page 3. The first one and half paragraphs are not referenced. Please provide appropriate references. 
Page 6 (counting from the title page), line 30, the experiment does not show directly that transgene silencing is piRNAs mediated. Please adjust the statement, or provide genetic support for the role of piRNAs/PIWI proteins. Same for line 37, no direct evidence is provided that ZAM silencing is mediated by flamenco. 
Page 6, line 56. Proximal to what? Please define. 
Page 7, line 29: how many ZAM derived reads do not map to piRNA clusters?
Discussion. Please discuss your results in context of Khurana et al Cell 2011.
Page 9, line 56, Fig S4D should be Fig 4D.
Page 9, line 60. If I understand the experiment of Fig 4d correctly, ZAM derived piRNAs could be produced by the transgene as well as the endogenous ZAM piRNA producing cluster, and these cannot be distinguished by sequencing. Thus the authors cannot unambiguously that significant amount of piRNAs were produced from ZAM-fraction of the transgene produces significant amount of piRNAs. Please explain or adjust the statement. 
Page 9, the genetics may be a bit complicated for non-drosophilists. Perhaps a scheme in the supplement would make this experiment more intuitive?
Page 10, line 56. Phidippo and pifo appear to be exclusively silenced by flamenco. Please provide a reference. 
Page 11, line 42. 'Several secondary mutations … were recovered'. Do the authors mean second site mutations, or additional (independent) mutations. Please define. Also, the origin of the different Rev lines is not completely clear to me. Perhaps here also a scheme in the supplement could help? 
Discussion. The authors propose several time that the germline can 'rapidly' control TEs by trapping them in piRNA clusters. I found rapidly a bit non-specific, and seem to be based on the analyses of the different Rev lines. But I can imagine that the insertion could have arisen multiple generations after the flamenco locus was mutagenized (is this still rapidly?). Is it possible to be more specific about the propose timing of the TE trapping?
Page 18, line 50. Why are different window size used for the different TEs? Please define. Also what steps are taken for sliding?
Fig. 2B. It would be useful to describe the other TEs (Pifo, etc) that are analyzed later in the manuscript. Also, genome coordinated on the x-axis would be useful. 
Legend to Fig. 2B. the legends suggests that antisense reads are in grey, but I think the antisense are in black in the plot. It may be useful to indicate the orientation of transcription of flamenco in the plot. 
Fig 3C and F and Fig 6B and E. separate sequence logos for sense and antisense may provide clearer logos. 
Figure 3. Have unique reads been used for the analyses of % reads with PPP, overlaps, etc. Please specify in the material and methods. 
Figure 5B and C are unique reads presented? Please specify in materials and methods. 
Figure 5A. It would be useful to provide the sequence logos in addition to the overlap plots.
Figure S1. Please show w[ir6] as this is the parental line to RevI.
Figures S3c. Please provide the data from w[ir6] as for comparison. 
Figure S5. Please also show the other TEs from Fig 5A for comparison.

**Authors’ response to reviewers**

***Reviewer #1****:*

*In their paper, Duc et al. investigate how Drosophila germline cells respond to the introduction of somatic follicular cell-specific ZAM endogenous retrovirus. The experiments are based on the observation that in Drosophila lines harboring a deletion within flamenco, a major piRNA cluster that is specifically expressed in somatic follicular cells, ZAM is de-repressed and invades germline genome. In the mutants, ZAM elements are inserted into a germline dual-strand piRNA cluster and corresponding piRNAs with the ping-pong signature are produced to silence ZAM in germline cells. These findings suggest that the germline can protect itself by trapping invading somatic-specific transposable elements (TEs) into germline piRNA clusters.*

*The mechanisms of transposon silencing in germ cells and silencing of invading genetic elements are very important for fertility and evolutionary history, yet they are incompletely understood. The Duc et al. study provides insight into a long-standing question of how invading somatic TEs are acted upon in the ovaries. The manuscript is of general interest and thus well within the scope of Genome Biology.*

*General remarks:*

*1. This work lacks information as to how germ cells initially respond to ZAM invasion and what might be the*

*consequences of ZAM invasion and then how germ cells recover from the damage caused by the invasion. The*

*RevI-H2 line the authors use in the paper was established nearly twenty years ago.*

Response: As it is mentioned in the main text and precise by the reviewer “The Rev line was first identified two

decades ago” and we agree with the reviewer that the initial response to *ZAM* invasion, what might be the

consequences of *ZAM invasion,* and how germ cells subsequently recover from the invasion-originated damage is a very interesting topic. We are currently investigating these different questions in an ongoing study. We are creating recombinant lines to investigate the initial response to *ZAM* invasion by creating a line with a recombined X chromosome which carries the *flamenco* allele with the large deletion but not the germline repressor inserted into a germline piRNA cluster. All these genetic analyses take time (several years) and cannot be included in this

manuscript.

*2. The authors conclude that ZAM-derived piRNAs are originated from a new ZAM insertion into a germline dualstrand piRNA cluster mainly because they display a ping-pong signature. However, strong evidence to support the conclusion is not present in the paper. The authors shall consider sequencing the RevI-H2 genome to identify ZAM insertions in germline piRNA clusters.*

Response: We think this comment is the result of our presentation of the genetic crosses not being clear enough,

thereby leading to a misunderstanding. The conclusion that the new *ZAM* insertion occurred into a germline piRNA cluster is mainly based on the genetic analysis and not on the ping-pong signature. Indeed, germline piRNAs are maternally deposited in the embryo and this transgenerational piRNA inheritance is required to trigger piRNA biogenesis in the progeny. Our genetic experiments showed that the unknown piRNA cluster that can silence the sensor transgene in the germline does not need maternal deposition of *ZAM*-derived piRNAs to be active since the pGFP-ZAM transgene is silenced in both ZMD and NZMD progenies. This is a proof that it is not a *de novo* piRNA cluster. We were probably not clear enough so we added additional text to set up the importance of this experiment on page 9 and a scheme describing genetic crosses and the two hypotheses in Fig. S4A.

Moreover, we are aware that besides the genetic crosses that strongly suggested the insertion of *ZAM* in an existing germline piRNA cluster, the previous version of the manuscript lacks other evidence. For this reason, we chose to look for the precise location of the new *ZAM* insertion present in an existing germline piRNA cluster on the X chromosome of RevI-H2 by deep sequencing of the RevI-H2 genome. Standard approaches to mapping TEs in short read genome sequencing data did not enable us to localize new *ZAM* insertions in piRNA clusters. Hence, we analyzed the genome sequencing data of RevI-H2 using a different method, which aims to identify chimeric reads (which were not mappable on the reference genome) containing both *ZAM* sequence and genomic sequence to specifically identify new *ZAM* insertions within repeated sequences. The *ZAM* sequences were then stripped off from these chimeric reads and the resulting sequences mapped to the *D. melanogaster* genome Release 6.03. This method allowed us to identify a new *ZAM* insertion within a dual-strand piRNA cluster next to the centromere of the X chromosome. We also displayed PCR results which confirm the presence of a *ZAM* insertion within this piRNA cluster. We show that this *ZAM* insertion is absent from the stable lines wIR6, ISO1A and w1118. We sequenced the PCR product from RevI-H2 and the sequence definitely confirmed the presence of a new ZAM insertion within the dual-strand piRNA cluster as predicted from DNA-seq data. These results are now described in the main text and presented in Fig. S4F-H.

*3. The novelty of the present study has been somewhat dampened by previous studies by others including*

*Theurkauf, Zhao Zhang and their colleagues examining how the host germ cells initially respond to an invasion and subsequently tame TEs. The authors should cite their papers (Khurana et al., Cell 2011; Moon et al., Dev Cell 2018) and discuss their work.*

Response: We agree with the reviewer and now discuss how our study compares to these two papers in the

discussion page 16.

*Some other comments:*

*4. Figure 3F, 6B, and 6E do not appear to show a bias for adenine at the tenth position (10A).*

Response: Logos presented in these figures were done with all *ZAM*-derived piRNAs and not only with the *ZAM*derived piRNAs having ping-pong partners. This is the reason why there is not a very strong bias for adenine at the tenth position. We now added logos done for the *ZAM*-derived piRNAs harboring ping-pong partners in Fig. S2A-B and S6C-F. It is then much easier to see that the sense *ZAM*-derived piRNAs harboring ping-pong partners have a strong bias for adenine at the tenth position (Fig. S2A, S6C, S6E) while the antisense ones have a strong uridine bias at the 5’ end (Fig. S2B, S6D, S6E).

*5. It would be kind to the reader if Figure 2B indicated transcription direction and size scale.*

Response: The transcription direction and size scale have been added in Fig. 2B.

*6. References are rough and sloppy. For example, many of papers in References contain information on publishers but many others do not.*

Response: We corrected the references.

*7. The authors should define "PPP" in the main text but not in figure legend.*

Response: We added details in the text. To be more precise, we now wrote: “Moreover, in RevI-H2 samples, 34% of the ZAM-derived piRNAs possessed ping-pong partners (PPP), *i.e.* piRNAs which present a 10-nt 5’-overlap between sense and antisense ZAM-derived piRNAs (Fig. 3H).

***Reviewer #2****:*

*In this manuscript, Duc et al describe an interesting case in which the retrotransposon ZAM is trapped into a germline cluster, leading to the production of ping-pong derived piRNAs, presumably to protect te germline*

*from further ZAM integrations. This work fits the hypothesis that piRNA clusters are a repository of past transposon invasions that provide piRNA-based protection from transposon activity/mobility. In this model, new transposon invasions would be uncontrolled until an insertion into a piRNA clusters initiates a piRNA-based responses.*

*Although this model is widely accepted, it has been hard to capture the event of novel transposon insertions into*

*clusters. Yet, there is some experimental evidence for this model, also in flies (e.g. Khurana et al Cell 2011, which to my surprise was not discussed in the current manuscript).*

*The current manuscript differs from previous work in that ZAM is a somatic transposon that is repressed in somatic follicular cells by the flamenco locus. The authors study a fly line in which part of the flamenco locus is deleted, including the part that produces ZAM piRNAs, which lead to ZAM invasion of the germline. Hence, the transposon invasion in this manuscript is from an endogenous somatic source.*

*I found this a well-written and interesting manuscript. See below for my comments and suggestions.*

Response: We really appreciate the positive comments on the various aspects of our work.

*Major points:*

*1. Support for a new ZAM insertion is rather indirect and mostly based on genetics, through the detection of ZAMderived ping-pond dependent piRNAs and repression of a GFP-ZAM transgene. The manuscript would be stronger if the authors could show direct evidence of a new ZAM insertion. As the authors propose that the insertion occurred in a pre-existing cluster (on chromosome X), a candidate PCR based approach or inverse PCR may work, or otherwise a deep-sequencing based approach could be explored. This would also help to define whether the insertions in the different Rev lines (page 12) are similar or different.*

Response: We agree with the reviewer, this limit being also pointed out by reviewer 1. As detailed in the answer to reviewer 1 ("general remarks" point 2), we now mapped, cloned and sequenced the new *ZAM* insertion.

*2. Several instances quantitative claims are made based on deep sequencing data, which is not a reliable quantitative method. As far as I see, the small RNA analyses are derived from a single library and thus prone to*

*biological noise (even if the libraries are made from material from multiple animals). Multiple independent small*

*RNA libraries should be analysed anywhere quantative conclusions are drawn.*

Response: We agree with the reviewer but since we sequenced RevI-H2 ovaries from 100 individuals and the

results are clearly different from the piRNA pattern observed in wIR6 (for which we provide another sequencing

dataset below, Complementary Fig. 1), we do not think that it is necessary to sequence the RevI-H2 library again.

In addition, we also sequenced the RevII-7 line which was recovered from the RevI-H2 line as well as the progeny of a cross between the RevI-H2 female and the control line harboring the *pGFP-ZAM* transgene and similar results and pattern as the RevI-H2 line were obtained for *ZAM*-derived piRNA (results for *ZAM*-derived piRNAs are presented below in Complementary Fig. 2 but not in the manuscript not to dilute the main message).

The observed effects on *ZAM*-derived piRNAs in different Rev lines are therefore very unlikely to be caused by biological noise. In addition, we proved that the new germline *ZAM*-derived piRNAs are functional because they are able to silence the p*GFP-ZAM* transgene in a piRNA pathway-dependent manner (new results presented in the revised manuscript). Nevertheless, we revised our manuscript to remove quantitative claims (16 times more have been removed) when they cannot be justified and now drew more qualitative conclusions from the data.

Complementary Figure 1: **a** Density profile of unique piRNAs from the wIR6 line that map to the *flamenco* piRNA

cluster. Sense and antisense reads are presented in black and grey, respectively. Almost no antisense reads map to the flamenco piRNA cluster. *ZAM* location in flamenco is displayed by a purple box. The *flamenco* deletion distal break-point in RevI-H2 is indicated by a red arrow and the sense of transcription by a black arrow. The count of piRNA reads per million (RPM) mapping the non-deleted region of *flamenco* is indicated below. **b** Density profile of *ZAM*-derived piRNAs along the 8.4kb *ZAM* sequence in wIR6 ovaries (allowing up to 3 mismatches). Sense and antisense reads are represented in black and grey, respectively. *ZAM* organization is displayed above the profile. LTR, long terminal repeats. **c** Bar diagram showing the total amount of *ZAM*-derived piRNAs produced in wIR6 ovaries quantified from the profile shown in *b*. **d** Logo of nucleotide bias for the first ten positions in *ZAM*-derived piRNAs produced in wIR6 ovaries. The nucleotide height represents its relative frequency at that position. **e** Histogram showing the percentage of 5'-overlap between sense and antisense *ZAM*-derived piRNAs (23-29nt) in wIR6 ovaries. The proportion of 10-nt overlapping pairs is in red and the Z-score is indicated.

Complementary Figure 2: **a** Density profile of *ZAM*-derived piRNAs along the 8.4kb *ZAM* sequence in ovaries of the progeny of RevI-H2 females crossed to the control line harboring the p*GFP-ZAM* transgene (allowing up to 3 mismatches). Sense and antisense reads are represented in black and grey, respectively. *ZAM* organization is displayed above the profile. LTR, long terminal repeats. **b** Bar diagram showing the total amount of *ZAM*-derived piRNAs produced in ovaries of the progeny of RevI-H2 females crossed to the control line harboring the p*GFP-ZAM* transgene, quantified from the profile shown in *a*. **c** Histogram showing the percentage of 5'-overlap between sense and antisense *ZAM*-derived piRNAs (23-29nt) in ovaries of the progeny of RevI-H2 females crossed to the control line harboring the p*GFP-ZAM* transgene. The proportion of 10-nt overlapping pairs is in red and the Z-score is indicated. **e-f** Logo of nucleotide bias for the first ten positions in sense (*e*) and antisense (*f*) *ZAM*-derived piRNAs with ping-pong partner (PPP) produced in ovaries of the progeny of RevI-H2 females crossed to the control line harboring the p*GFP-ZAM* transgene. The nucleotide height represents its relative frequency at that position.

Table: This table summarize details for the various samples for which small RNA sequencing was performed.

| Line | Number of flies per sample | Number of reads |
| --- | --- | --- |
| w^IR6^ | 100 | 27,727,109 |
| RevI-H2 | 100 | 17,072,790 |
| RevI-H3 | 60 | 13,788,263 |
| RevII-7 | 60 | 9,276,894 |
| Early embryos of RevI-H2 |  | 11,538,833 |
| Progeny of RevI-H2 females crossed to the control line harboring the p*GFP*-*ZAM* transgene | 100 | 13,957,858 |
| Progeny of wIR6 females crossed to the control line harboring the p*GFP-ZAM* transgene | 100 | 10,451,167 |
| Second sample for w^IR6^ | 60 | 13,576,009 |

*3. Page 9, the authors propose that ZAM could have inserted into a pre-existing cluster or could form a novel cluster. However, the experiment performed does not directly discriminate between both possibilities, yet a strong conclusion is made that ZAM inserted into a pre-existing cluster (line 47). Directly mapping the new insertion (see point 1) would address this concern. Should that not be successful, the statement needs to be adjusted.*

Response: As noted above in the answer to reviewer 1 ("general remarks" point 2), we think this comment is the result of our presentation of the genetic crosses not being clear enough, thereby leading to a misunderstanding. As mentioned above in the answer to the comment 1 of the reviewer, we now improved description of this experiment in the main text and in Fig S4A with a schema, and provided evidence of the new *ZAM* insertion based on genomic, PCR and sequencing data.

*4. Fig.1. A control experiment with a GFP transgene without a ZAM sequence in the UTR would be needed as a reference.*

Response: We added the control experiment which consist of the *GFP* transgene from which the *ZAM* sequence was excised (named p*GFP*). In RevI-H2 germline cells, the p*GFP* transgene is expressed while the p*GFP-ZAM* one is silenced. In w^IR6^ somatic cells, the p*GFP* transgene is expressed while the p*GFP-ZAM* one is silenced. These experiments clearly show that the *ZAM* sequence is indeed the target of this silencing mechanism. Results are presented in Fig. S3H. In the manuscript, we added the following statement “When the ZAM sequence was excised upon recombination between the flanking FRTs giving rise to the p*GFP* transgene, GFP is strongly expressed in both RevI-H2 and w^IR6^ somatic and germline cells indicating that the ZAM sequence in the fusion transcript is responsible for GFP repression (Fig. S3H).”

*Minor points*

*Page 3. The first one and half paragraphs are not referenced. Please provide appropriate references.*

Response: We provided appropriate references.

*Page 6 (counting from the title page), line 30, the experiment does not show directly that transgene silencing is piRNAs mediated. Please adjust the statement, or provide genetic support for the role of piRNAs/PIWI proteins.*

Response: We now provided the genetic experiment to confirm that transgene silencing is piRNAs mediated. We knocked down the germline expression of Aub or AGO3 (using the female germline-specific *nanos*-GAL4 driver together with the corresponding UAS-shRNA transgenic lines produced by the TRiP project, they are named Aub-KD and AGO3-KD) and we monitored *GFP* expression of the p*GFP-ZAM* sensor transgene in a RevI-H2 genetic background. Actually, we showed that the transgene is strongly expressed in the germline of both Aub-KD and AGO3-KD (Fig. S3I-J), confirming that the transgene is silenced by a piRNA-mediated mechanism. These results are now described in the main text and presented in Fig S3I-J.

*Same for line 37, no direct evidence is provided that ZAM silencing is mediated by flamenco.*

Response: We added the reference that proved that *ZAM* silencing is mediated by *flamenco* (Mével-Ninio *et al.,* 2007).

*Page 6, line 56. Proximal to what? Please define.*

Response: Genetically the term "proximal" region is always the region closest to the centromere (in opposition to "distal"). We added this definition in the manuscript.

*Page 7, line 29: how many ZAM derived reads do not map to piRNA clusters?*

Response: Among the 17,798 *ZAM-*derived mappers (all ZAM mappers, allowing up to 3 mismatches) in w^IR6^, 15,646, i.e. 87.9% match piRNA clusters as defined by Brennecke et al., 2007 without mismatch. Thus 12.1% of *ZAM* derived reads did not map to piRNA clusters.

*Discussion. Please discuss your results in context of Khurana et al Cell 2011.*

Response: We agree with the reviewer and now discuss this paper in the discussion.

*Page 9, line 56, Fig S4D should be Fig 4D.*

Response: We replaced "Fig. S4D" with "Fig. 4D" at this location in the manuscript.

*Page 9, line 60. If I understand the experiment of Fig 4d correctly, ZAM derived piRNAs could be produced by the transgene as well as the endogenous ZAM piRNA producing cluster, and these cannot be distinguished by sequencing. Thus the authors cannot unambiguously that significant amount of piRNAs were produced from ZAM-fraction of the transgene produces significant amount of piRNAs. Please explain or adjust the statement.*

Response: We agree and we have modified this statement as proposed by the reviewer. We now wrote: "... , while a significant amount of piRNAs mapped to the *ZAM* fragment in the ZMD progeny (Fig. 4D; right panel)."

*Page 9, the genetics may be a bit complicated for non-drosophilists. Perhaps a scheme in the supplement would make this experiment more intuitive?*

Response: We added a scheme in Fig. S4A to explain genetic crosses and both hypotheses.

*Page 10, line 56. Phidippo and pifo appear to be exclusively silenced by flamenco. Please provide a reference.*

Response: We changed the statement to “mainly silenced by *flamenco”*. Indeed, in the Fig 5B et 5C, the Pifo and *Phidippo*- derived piRNAs disappear almost completely strongly suggesting that they are nearly exclusively produced by *flamenco*.

*Page 11, line 42. 'Several secondary mutations … were recovered'. Do the authors mean second site mutations, or additional (independent) mutations. Please define. Also, the origin of the different Rev lines is not completely clear to me. Perhaps here also a scheme in the supplement could help?*

Response: We now referred to the article describing all the Rev lines from Desset el al, 1999 and to the review where a scheme, explaining how these different Rev lines were derived, is presented (Goriaux et al, 2014).

*Discussion. The authors propose several times that the germline can 'rapidly' control TEs by trapping them in piRNA clusters. I found rapidly a bit non-specific, and seem to be based on the analyses of the different Rev lines. But I can imagine that the insertion could have arisen multiple generations after the flamenco locus was mutagenized (is this still rapidly?). Is it possible to be more specific about the propose timing of the TE trapping?*

Response: We are currently investigating all these questions in a present study. We are creating recombinant lines to investigate the initial response to *ZAM* invasion by creating a line with a recombined X chromosome which carries the *flamenco* allele with the large deletion but not the germline repressor inserted into a germline piRNA cluster. However, all these genetic analyses take time (several years) and are not far enough to be included in this manuscript.

RevI-H3 and RevI-H2 lines were isolated two years after the mutagenesis. Hence, we considered the phenomena rapid regarding the evolution time scale of a species, but we agree with the reviewer that “rapidly” is a bit non-specific so we removed it from the discussion.

*Page 18, line 50. Why are different window size used for the different TEs? Please define. Also what steps are taken for sliding?*

Response: We chose a fixed number of windows per analyzed sequence, meaning that the window size depends on the length of the analyzed sequence. There are no sliding windows here. We added the following sentence in the Methods: “The window size … and depended of the TE size”.

*Fig. 2B. It would be useful to describe the other TEs (Pifo, etc) that are analyzed later in the manuscript. Also, genome coordinated on the x-axis would be useful.*

Response: We provided location on *flamenco* for the TEs described in this manuscript as well as the X chromosome coordinates of *flamenco* in Fig. S1A.

*Legend to Fig. 2B. the legends suggests that antisense reads are in grey, but I think the antisense are in black in the plot. It may be useful to indicate the orientation of transcription of flamenco in the plot.*

Response: We agree with the reviewer that we did not give enough details in the legend. Most of the reads that map to *flamenco* are sense reads. We added the following sentence in Fig.S2B legend “Almost no antisense reads map to the flamenco piRNA cluster.” to be more precise. We added the transcription direction in Figure 2B.

Because most of TEs inserted into the *flamenco* locus are in the opposite orientation to the *flamenco* transcription, most of the piRNA which are produced by flamenco are sense to flamenco and antisense to TEs. Fig.2B describes the flamenco piRNAs thus they are sense (with respect to flamenco transcription).

*Fig 3C and F and Fig 6B and E. separate sequence logos for sense and antisense may provide clearer logos.*

Response: We now added logos done for the *ZAM*-derived piRNAs harboring ping-pong partners in the Fig. S2A-B. It is then much easier to see that the sense *ZAM*-derived piRNAs harboring ping-pong partners have a strong bias for adenine at the tenth position (Fig. S2A, S6C, S6E) while the antisense ones have a strong uridine bias at the 5’ end (Fig. S2B, S6D, S6E).

*Figure 3. Have unique reads been used for the analyses of % reads with PPP, overlaps, etc. Please specify in the material and methods*.

Response: We provide further details in the Methods and add in the text “based on piRNAs mapping to the analyzed TE (0 to 3 mismatches)” and “mapping to the analyzed TE (0 to 3 mismatches)”.

*Figure 5B and C are unique reads presented? Please specify in materials and methods.*

Response: We provide further details in the text and add in the legend “using all piRNAs mapped to the corresponding TE allowing up to 3 mismatches”.

*Figure 5A. It would be useful to provide the sequence logos in addition to the overlap plots.*

Response: We provide logos for sense and antisense reads with PPP in Fig. S5A.

*Figure S1. Please show w[ir6] as this is the parental line to RevI.*

Response: We now provide in Figure S1B the small RNA read alignment on *flamenco* for the w^IR6^ line instead of Iso1A as the control line.

*Figures S3c. Please provide the data from w[ir6] as for comparison.*

Response: Since all piRNAs in early embryos are coming from the germline of the mother and no *ZAM*-derived piRNAs are produced in the germline of w^IR6^ females, we did not sequence small RNAs of w^IR6^ early embryos but we are prepared to do so if this should be considered as an essential point.

*Figure S5. Please also show the other TEs from Fig 5A for comparison.*

Response: We added the piecharts for Adoxo, Idefix, Gedeo, Vatovio in Fig. S5D.

**Second round of review**

**Reviewer 1**

On the whole it has been improved and the authors have addressed most of the reviewers concerns. There are no further technical concerns about the content and presentation of the manuscript in its current form. 

However, I found that to my surprise, the authors did not cite the paper of Gunawardane LS et al. for the ping-pong cycle (page 3 line 57). The authors should cite the paper. 

Gunawardane LS, Saito K, Nishida KM, Miyoshi K, Kawamura Y, Nagami T, Siomi H, Siomi MC. A Slicer-mediated mechanism for repeat-associated siRNA 5′ end formation in Drosophila. Science 2007. 315: 1587-1590.

**Reviewer 2**

In this revised manuscript, the authors have addressed all my comments. Most importantly, by genome-resequencing, they managed to identify the novel ZAM transposon insertion in a germline cluster, providing further evidence for their conclusion (and title). 
I congratulate the authors on a beautiful paper.
